# Low bone density and high morbidity in patients between 55 and 70 years with displaced femoral neck fractures: a case-control study of 50 patients vs 150 normal controls

**DOI:** 10.1186/s12891-019-2732-8

**Published:** 2019-08-14

**Authors:** Stefan Bartels, Jan-Erik Gjertsen, Frede Frihagen, Cecilia Rogmark, Stein Erik Utvåg

**Affiliations:** 1 Department of Orthopedic Surgery, Akershus University Hospital, Lørenskog, Norway; 2 Norwegian Hip Fracture Register, Department of Orthopedic Surgery, Haukeland University Hospital, Bergen, Norway; 3 Department of Clinical Medicine, University of Bergen, Bergen, Norway; 4 Department of Orthopedic Surgery, Oslo University Hospital, Oslo, Norway; 5 Department of Orthopedics, Skåne University Hospital, Lund University, Malmö, Sweden; 6 Institute of Clinical Medicine, University of Oslo, Oslo, Norway

**Keywords:** Femoral neck fracture, Bone mineral density, Comorbidity, Osteoporosis

## Abstract

**Background:**

A displaced femoral neck fracture (FNF) in patients 55-70 years is a serious injury with a high risk of treatment failure and the optimal surgical treatment remains unclear. We aimed to describe characteristics of fracture patients compared to a sample from the normal population.

**Methods:**

Fifty patients aged 55-70 years with a displaced FNF were gender- and age- matched with a control group of 150 persons without a hip fracture using computergenerated randomization and the Norwegian National Population Register. To reduce the risk of spurious selection bias, the sample size of the control group was trebled compared to the fracture group. Dual-energy x-ray absorptiometry (DXA) was performed. Demographics and hip function (Harris Hip Score, Oxford Hip Score, and Hip Dysfunction and Osteoarthritis Outcome Score) were collected.

**Results:**

There were more than 75% women in both groups. The mean age was 64.5 years in the fracture group and 65.1 in the control group. Results for DXA measured for lumbar spine, total hip and the femoral neck showed that patients with displaced FNF were significantly more osteoporotic. Fracture patients had significantly lower body mass index, higher Charlson comorbidity index (CCI), and higher ASA (American Society of Anesthesiologists) score than the control group. No clinically relevant differences in hip function were found. There were 48% smokers in the fracture group compared to 10% in the control group. The odds ratio for obtaining a displaced FNF was high if the patients suffered from osteoporosis, smoked or had several comorbidities.

**Conclusions:**

This study showed that patients aged 55-70 years with a displaced femoral neck fracture had lower bone density and higher comorbidity compared with a gender- and age-matched population without femoral neck fractures. This suggests that this patient group is epidemiologically similar to older patients with femoral neck fractures.

## Background

Regardless of age, a displaced femoral neck fracture (FNF) is a severe injury and will almost always require hospitalization and surgery [1]. Patients with these fractures have a high risk of subsequent surgical complications, reduced function, hip pain and reduced health-related quality of life. The health economic aspect is a great challenge, even though the overall incidence of hip fractures has decreased in recent decades [1–7]. The literature on elderly patients older than 70 years with displaced FNFs is extensive and arthroplasty is clearly recommended as the treatment of choice [8–14]. The middle-aged patient group aged 55-70 years is less well described and the treatment for displaced FNFs is still controversial [11–13]. These patients are probably still working and demand a high level of activity. A Norwegian study has reported the overall hip fracture incidence in this particular age group to be 92 per 10 000 (53 women and 39 men) in the period 2009-2013 [15]. We found a specific incidence of about 6.1 fractures for both genders per 10 000 for displaced FNFs in patients 55-70 years in 2017 in Norway [16, 17]. Most of these FNFs are caused by a low-energy trauma, and the patients often have other diseases and factors, such as medication (steroids, anti-epileptic medication), alcoholism, other substance abuse, or osteoporosis, all of which may increase the risk of complications, including revision surgery [18–20]. Studies including bone density at the time of fracture are rare and often described a more geriatric population [21]. For patients under 60 years of age, internal fixation (IF) is usually recommended, as many surgeons endeavor to prevent replacement of the hip joint [12, 13, 22]. Studies investigating outcome after FNF in patients younger than 70 years have found a high risk of reoperation after IF due to mechanical failure, non-union or avascular necrosis [22–24]. Most of the investigated patients in this age group had symptomatic comorbidities and the 1-year mortality has been reported to be as high as 15% [24]. This may indicate that many hip fracture patients under 70 years of age are more osteoporotic and frailer than individuals at the same age in the general population. Thus, their fractures may beneficially be treated mainly by arthroplasty, as in patients older than 70 years.

The aim of the present study was to describe differences in bone density and morbidity at the time of injury between a group of 50 patients aged 55-70 with a native intracapsular displaced FNF compared with a gender- and age- matched cohort of 150 participants from the general population without a fracture.

## Methods

This pragmatic gender- and age- matched case-control included 50 consecutive patients aged 50-70 years presenting with a low-energy displaced FNF in the native hip joint and belonging to the catchment area of Akershus University Hospital, Norway from December 2013 to November 2017. The control group consisted of 150 participants from the population in the same catchment area. The Department of Data and Analytics at Akershus University Hospital was responsible for the recruitment of this group using computergenerated randomization lists and both the National Population Register and the unique national identification number assigned for each inhabitant in Norway. Patients with cognitive impairment were not included in either group. Controls were matched to the fracture group by loose matching [25]. For matching we divided the patients into three age groups for both genders (55-59, 60-64, 65-69 years) using the number of cases in the fracture group as the base. The tripled size of the control group was chosen to achieve statistical power [26]. Three hundred and forty-one potential participants were invited by mail, and one hundred and ninety-one persons declined or did not attend the agreed appointment. Information from the FNF patients was collected before discharge. DXA was performed postoperatively before discharge or within at least 6 weeks after injury. No anti-osteoporotic medication was given to the fracture patients group before DXA was performed. Interviews, questionnaires, and DXA for the control group were performed between November 2016 and June 2017 during a single outpatient appointment. All participants in the control group and in the fracture group signed an informed consent form.

The following variables were registered: age, gender, height and weight (measured in conjunction with DXA using a standard scale and a stadiometer), current smoking status, Charlson comorbidity index (CCI), American Society of Anesthesiologists (ASA) score and presence of diabetes [27, 28]. Furthermore, the Harris Hip Score (HHS), Oxford Hip Score and the Hip Dysfunction and Osteoarthritis Outcome Score (HOOS) were recorded [29–31]. For patients with displaced FNF, we asked for specific hip function scores the last week before the current fracture. Body Mass Index (BMI) was calculated as weight/height^2^ in kg/m^2^. Each participant in the control group and patients in the fracture group underwent a DXA measurement using lumbar spine (L1-L4), total hip and femoral neck. Five patients in the fracture group could not undergo DXA measurement in the hip, due to implants after recent fracture care and from previous surgery unrelated to the recent injury in the contralateral side.

The DXA scan was used to determine bone mineral density (BMD) measured in the lumbar spine (L1-L4), total hip and the femoral neck. All BMD measurements were performed by two independent DXA technicians on the same DXA scanner (Lunar iDXA™ GE Healthcare Lunar, Global Headquarters, P O Box 7550, Madison, Wisconsin 53707-7550, USA).

The definition of osteoporosis was that used by the World Health Organization, where a T-score ≤ - 2.5 SD is osteoporosis, a T-score > - 2.5 - < - 1 SD is osteopenia and T-score ≥ - 1 SD is normal bone [32].

### Statistical analysis

Continuous variables are presented as mean values and categorical variables were summarized by the number of subjects and percentage in each category. We used the independent samples t-test for continuous variables with normal distribution, and the Mann-Whitney U-test for non-normally distributed outcomes. The Pearson Chi-square test was used for categorical variables. All tests were two-sided and results were considered statistically significant at a 5% level. To identify risk factors for a femoral neck fracture we used a logistic regression model with independent variables. The Odds Ratios (ORs) and the 95% Confidence Intervals (CIs) were reported. The analyses were performed using IBM SPSS, version 25.0 (IBM Corp., Armonk, NY, USA).

## Results

### Comorbidity

**Fig. 1:**
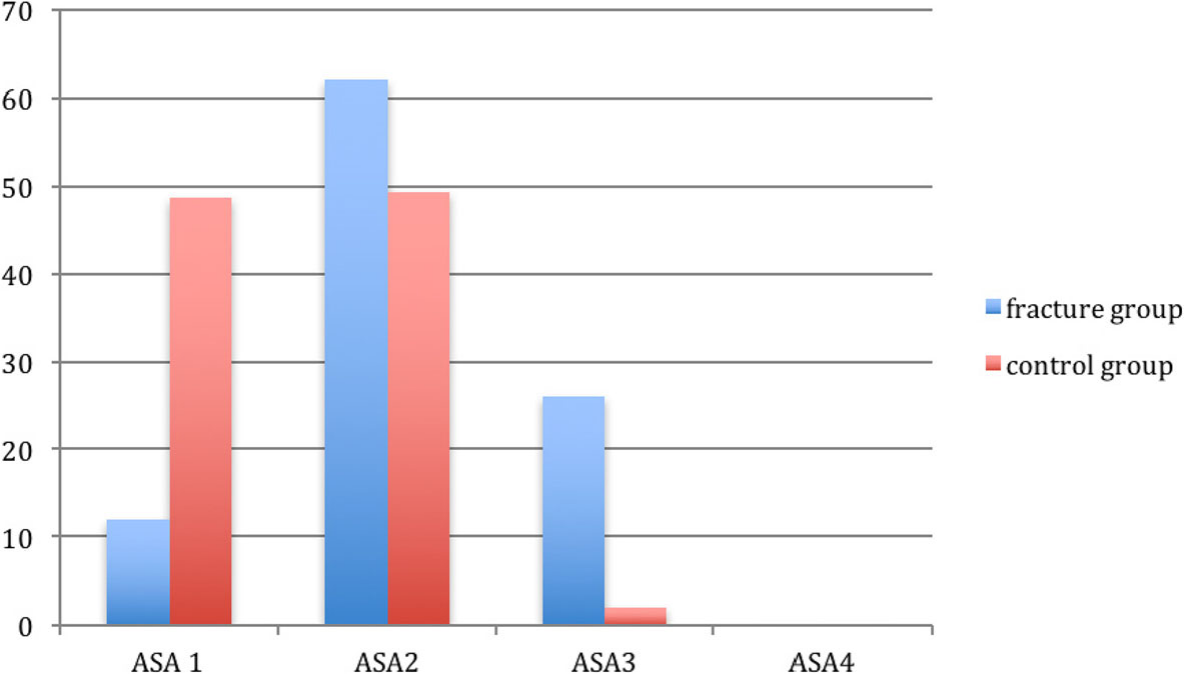
Differences in comorbidity presented by ASA classification in % for both groups

We found more comorbidities (higher CCI score and more patients with ASA class 2-3) in the fracture group (Table 1) (Fig. 1). Patients in the fracture group had lower BMI and there were more smokers in this group.

**Table 1:** Baseline characteristics according to the two different groups

	Fracture group *n* = 50	Control group *n* = 150	*p*- values
Mean age, years (95% CI)	64.5 (63.5-65.6)	65.1 (64.4-65.7)	0.677 ^a^
Gender, w/m (%)	38 (76%) / 12 (24%)	116 (77.3%) / 34 (22.7%)	0.846 ^b^
Mean BMI (kg/m^2^) (95% CI)	24.2 (23.1-25.4)	26.7 (26.0-27.4)	0.001^c^
Diabetes mellitus, n (%)	5 (10%)	5 (3.33%)	0.061^b^
Smoking, n (%)	24 (48%)	15 (10%)	< 0.001^b^
Mean Charlson Comorbidity Index Score total (95% CI) n (%)	2.64 (2.39-2.89)	2.14 (2.04-2.24)	0.001^b^
1	3 (6%)	19 (12.7%)	
2	20 (40%)	94 (62.7%)	
3	21 (42%)	34 (22.7%)	
4	4 (8%)	3 (2%)	
5	2 (4%)	0	
ASA score, n (%)^d^			< 0.001^b^
1	6 (12%)	73 (48.7%)	
2	31 (62%)	74 (49.3%)	
3	13 (26%)	3 (2%)	
4	0	0	

^a^independent samples t-test

^b^Pearson Chi-square test

^c^Mann-Whitney U-test

^d^American Society of Anesthesiologists

### Hip function

**Fig. 2:**
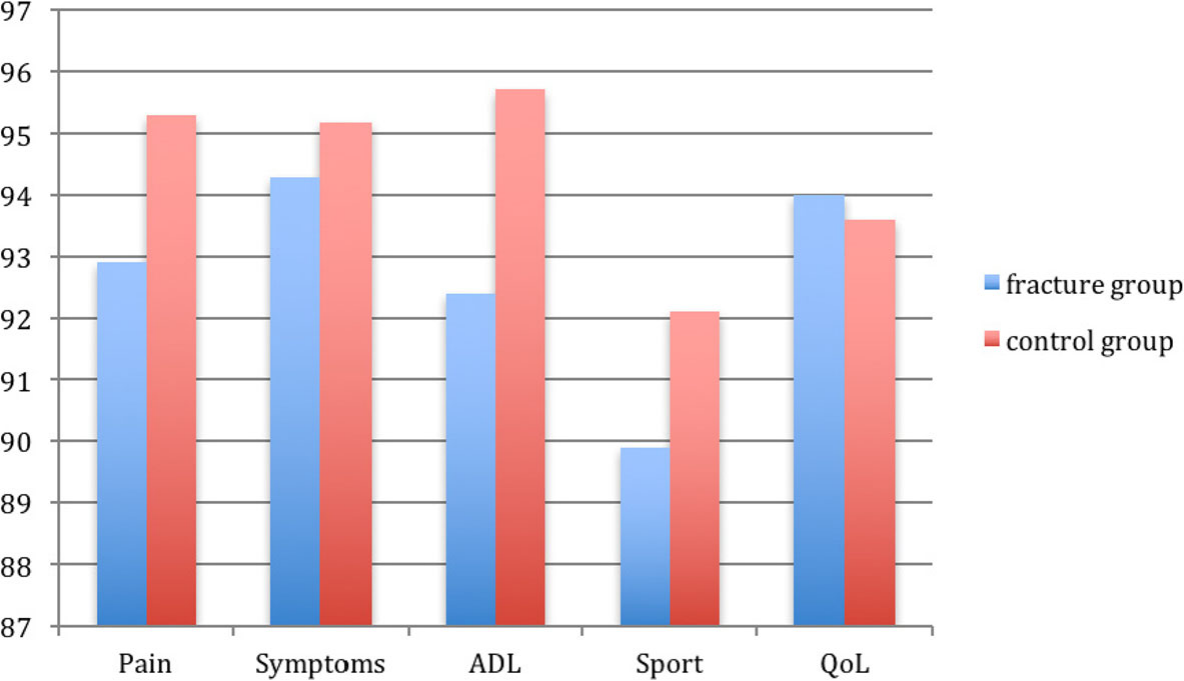
Differences in hip function presented by HOOS for both groups and before fracture

No significant differences in hip function using the Oxford Hip Score and HOOS could be found, but the control group reported better HHS (97.1) compared to the fracture group (93.6) (Table 2) (Fig. 2).

**Table 2:** Differences in hip function between cases (before fracture) and controls

	Fracture group *n* = 50	Control group *n* = 150	*p*-values
Mean Harris Hip Score (95% CI)	93.6 (91.0-96.3)	97.1 (95.8-98.4)	< 0.001^a^
Mean Oxford Hip Score (95% CI)	46.2 (44.8-47.2)	46.0 (45.4-47.0)	0.173 ^a^
Mean HOOS (95%CI) Pain	92.9 (89.1-96.7)	95.3 (93.5-97.1)	0.226 ^a^
Symptoms	94.3 (90.6-97.7)	95.2 (93.4-97.0)	0.226 ^a^
ADL	92.4 (87.3-97.5)	95.7 (93.9-97.5)	0.244 ^a^
Sport	89.9 (83.6-96.2)	92.1 (89.3-94.8)	0.739 ^a^
QoL	94.0 (89.7-98.4)	93.6 (90.9-96.4)	0.792 ^a^

^a^Mann-Whitney U-test

### Bone mineral density

**Fig. 3:**
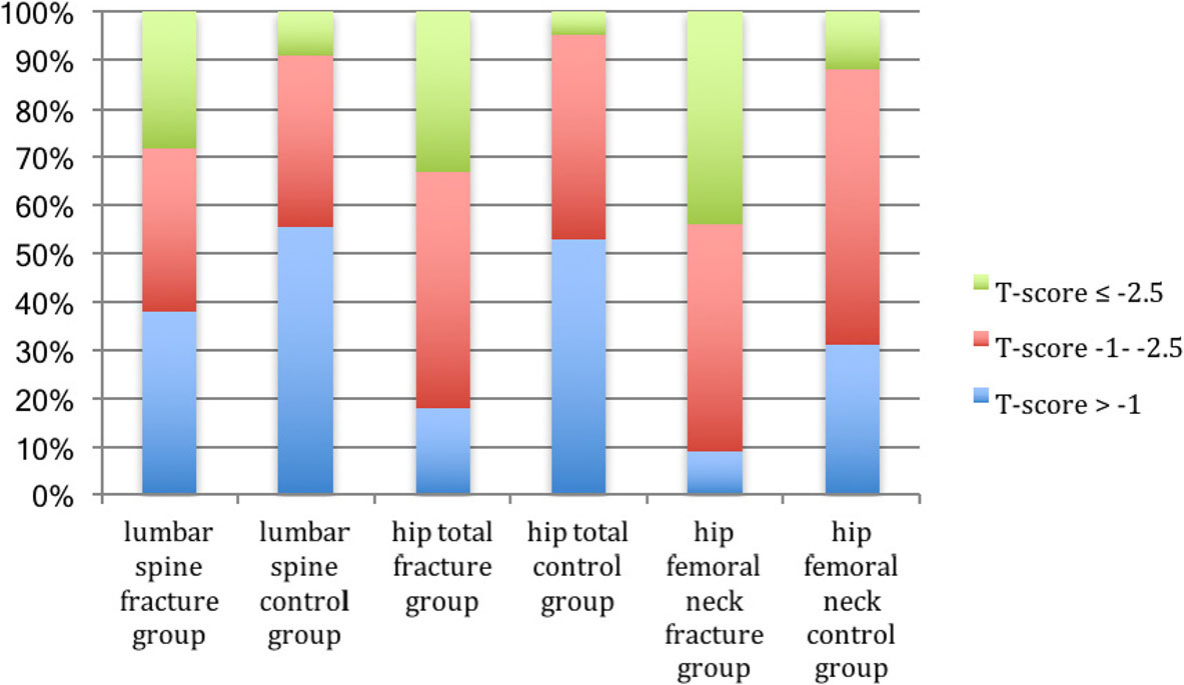
T-score values for fracture and control group

BMD measurements were performed for all participants in the control group. Five participants in the fracture group had implants in both hips, making measurements in this area impossible. There were more patients with osteopenia and osteoporosis in the fracture group compared to the control group both when comparing results from lumbar spine, total hip, and the femoral neck (Table 3) (Fig. 3).

**Table 3:** Differences in DXA-measures between 50 patients with FNF and the control group

Dual-energy X-ray absorptiometry	Fracture group *n* = 50/45 hips *n* (%)	Control group *n* = 150 *n* (%)	*p*-values
Lumbar spine T-score			0.003 ^a^
T-score >-1	19 (38%)	83 (55%)	
T-score - 1- -2.5	17 (34%)	53 (35%)	
T-score ≤ -2.5	14 (28%)	14 (9%)	
Hip total T-score ^b^			< 0.001 ^a^
T-score >-1	8 (18%)	79 (53%)	
T-score - 1 - -2.5	22 (49%)	63 (42%)	
T-score ≤ -2.5	15 (33%)	8 (5%)	
Hip femoral neck T-score ^b^			< 0.001 ^a^
T-score >-1	4 (9%)	47 (31%)	
T-score- 1 - -2.5	21 (47%)	85 (57%)	
T-score ≤ -2.5	20 (44%)	18 (12%)	

^a^Pearson Chi-square test

^b^5 patients with implants after fracture care in both hips, making measurements in the hips impossible

### Risk analysis

A multivariate logistic regression analysis for unadjusted variables showed statistically significant differences in the OR for smoking, presence of osteoporosis, and CCI score (Table 4). The adjusted OR for obtaining a femoral neck fracture was 6.7 for smokers compared to non-smokers, and 7.5 for participants with osteoporosis compared to participants with normal BMD.

**Table 4:** Logistic regression with Odd ratio obtaining displaced FNF for multivariate analyses and with adjustments

	Unadjusted			Adjusted ^a^		
	OR	95%CI	*p*-values	OR	95%CI	*p*-values
Smoking	8.3	3.8-17.9	< 0.001	6.8	2.8-16.1	< 0.001
Charlson Comorbidity Index Score	2.6	1.6-4.1	< 0.001	2.0	1.2-3.6	0.011
DXA femoral neck T-score > -1 normal bone	1		< 0.001	1		< 0.001
DXA femoral neck T-score- 1 - -2.5 osteopenia	3.1	1.0-9.6	0.049	2.1	0.6-6.9	0.236
DXA femoral neck T-score ≤ -2.5 osteoporosis	13.6	4.1-45.3	< 0.001	7.6	2.1-27.9	< 0.001

^a^adjusted for all other variables in the table in a multiple regression analysis model

## Discussion

The present study showed that patients aged 55-70 years with a displaced FNF were more osteoporotic and had more comorbidities compared to a gender- and age- matched control group. In addition, we found that fracture patients were more frequently smokers and had lower BMI. Thus, our data suggests that the risk of sustaining a displaced femoral neck fracture probably increases with the presence of osteoporosis, comorbidities and smoking. Regarding baseline hip function, the control group reported better scores in only one of the three hip scores used, suggesting that the difference was not clinically relevant.

There were statistically significantly more patients with low bone density in the fracture group. These findings agree well with other studies suggesting that young patients (< 50 years of age) with hip fracture due to low-energy injury suffer from early osteoporosis [18, 20]. We found more comorbidities in the fracture group, measured by both the CCI score and the ASA score. These findings are supported by another study reporting comorbidities as an important and determinant factor in non-elderly patients with hip fractures [20]. We found a significantly higher proportion of smokers in the fracture group, where almost 50% were smokers at the time of injury compared to 10% in the control group. This has also been confirmed by a meta-analysis identifying smoking as a risk factor for any kind of fracture and for hip fractures in particular [33]. Other authors have postulated smoking and diabetes mellitus as the strongest independent impact factors for increased hip fracture risk [34, 35]. Diabetes has previously been described as an important risk factor for hip fractures in both women and men [35–37]. A statistically significant difference in the presence of diabetes between the fracture group and the control group could not be found in our study. However, as we had a limited number of participants in our study, the power was probably insufficient to investigate this issue.

Lower BMI was found in the fracture group. Higher BMI has been argued to protect against a hip fracture, but probably not in this age group [38]. The lower BMI might also be associated with the larger number of smokers in the fracture group. Smoking is associated with negative influence on nutrition, which may result in lower BMI among smokers [33].

Our study suggests that the presence of osteoporosis and smoking were the strongest differences between the fracture group and the control group with a seven times higher OR for patients with displaced FNF when adjusting for smoking, comorbidity and BMD.

The main strength of our study is that the DXA measurements in the fracture group were performed at the time of injury, documenting the current bone status. Furthermore, we present results from a representative and relatively homogeneous gender- and age-matched population.

However, this study has limitations, as case-control studies are prone to bias, especially selection and recall bias. The number of fracture patients was limited, as the incidence of hip fractures in this age group is low [19]. The sample size was small and included both genders, recognizing the differences in risk factors and epidemiology of hip fracture in women and men [15, 18, 38, 39]. Exact power calculations were not performed, but tripling the number of controls was used to improve statistical strength [26]. We used a standard logistic regression model for a loose-matching strategy within pair correlation, approving that conditional logistic regression is the appropriate analytical method for matched case-control studies. We did not record several well-known risk factors for hip fractures and osteoporosis, such as alcohol consumption, calcium and vitamin D levels, differences in bone-associated comorbidities (e.g. malabsorption), hormonal deficits, or the use of specific medication influencing bone quality (steroids, anti-epileptic medication) [20, 34, 35, 39–41].

## Conclusions

Patients between 55 and 70 years with a displaced femoral neck fracture were more osteoporotic and have more comorbidities, than a comparable gender- and age- matched group from the general population without a femoral neck fracture. Our data indicate that these patients are frailer than expected and should probably not be compared with their peers of the same age. From a clinical perspective, patients aged 55-70 years may benefit from a similar treatment as those over 70 years, within a treatment algorithm considering biological age, individual factors, and medical challenges.

## Data Availability

Due to regulations from the Norwegian Data Inspectorate and according to Norwegian personal protections laws, publication of the complete dataset is not legal or appropriate.

## Abbreviations

95% CI: 95% confidence interval
ASA: American Society of Anesthesiologists
BMD: Bone mineral density
BMI: Body Mass Index
CCI: Charlson comorbidity index
DXA: Dual-energy x-ray absorptiometry
FNF: Femoral neck fracture
HHS: Harris Hip Score
HOOS: Hip Dysfunction and Osteoarthritis Outcome Score
IF: Internal fixation
OR: Odds ratio
